# The impact of data quality and source data verification on epidemiologic inference: a practical application using HIV observational data

**DOI:** 10.1186/s12889-019-8105-2

**Published:** 2019-12-30

**Authors:** Mark J. Giganti, Bryan E. Shepherd, Yanink Caro-Vega, Paula M. Luz, Peter F. Rebeiro, Marcelle Maia, Gaetane Julmiste, Claudia Cortes, Catherine C. McGowan, Stephany N. Duda

**Affiliations:** 1 Vanderbilt University School of Medicine, Nashville, TN USA; 2 Instituto Nacional de Ciencias Médicas y Nutrición Salvador Zubirán, Mexico City, Mexico; 3 Instituto Nacional de Infectologia Evandro Chagas, Fundação Oswaldo Cruz, Rio de Janeiro, Brazil; 4 Universidade Federal de Minas Gerais, Belo Horizonte, Brazil; 5 Les Centres GHESKIO, Port-au-Prince, Haiti; 6 Fundación Arriarán, University of Chile School of Medicine, Santiago, Chile

**Keywords:** Source data verification, Data audit, Data quality, Observational data, HIV, Latin America

## Abstract

**Background:**

Data audits are often evaluated soon after completion, even though the identification of systematic issues may lead to additional data quality improvements in the future. In this study, we assess the impact of the entire data audit process on subsequent statistical analyses.

**Methods:**

We conducted on-site audits of datasets from nine international HIV care sites. Error rates were quantified for key demographic and clinical variables among a subset of records randomly selected for auditing. Based on audit results, some sites were tasked with targeted validation of high-error-rate variables resulting in a post-audit dataset. We estimated the times from antiretroviral therapy initiation until death and first AIDS-defining event using the pre-audit data, the audit data, and the post-audit data.

**Results:**

The overall discrepancy rate between pre-audit and audit data (*n* = 250) across all audited variables was 17.1%. The estimated probability of mortality and an AIDS-defining event over time was higher in the audited data relative to the pre-audit data. Among patients represented in both the post-audit and pre-audit cohorts (*n* = 18,999), AIDS and mortality estimates also were higher in the post-audit data.

**Conclusion:**

Though some changes may have occurred independently, our findings suggest that improved data quality following the audit may impact epidemiological inferences.

## Background

Source document verification (SDV) is a strategy for research data quality assessment. Typically, SDV involves the partial (or complete) comparison of research study data against original source documents, such as study case report forms, patient clinical charts, laboratory reports, or electronic health records. This practice of data auditing allows investigators to verify data are entered according to study definitions, identify systematic issues with research data collection, and calibrate their confidence for making inferences based on study findings.

Concerns regarding data quality are magnified for studies using routinely collected observational data from international cohorts. Given that many HIV observational datasets were originally created for clinical or administrative purposes, data are susceptible to errors with respect to completeness and correctness [1]. Studies assessing HIV observational data quality in multiple international settings have identified data discrepancies and high error rates in key variables [2–5]. In an earlier audit of a subsample of records from a multiregional database of HIV clinical care sites, we found errors that were not flagged by computer-generated error reports and systematic inconsistencies in how data were entered [6].

Because SDV is resource-intensive – locating the original source documents, travel by external auditors to local sites, comparing source documents to the current research dataset, and recording discrepancies – it is becoming increasingly important to justify its expense. Many data audits assess data quality according to whether the error rate is above or below an arbitrary threshold [7]. However, as shown in clinical trial settings [8, 9], high error rates do not necessarily translate into invalid epidemiological inferences. In addition to quantifying error rates, the importance of the SDV process should be assessed by investigating potential improvements in data quality in the research network over time and the impact of errors on analyses and corresponding conclusions.

The analysis of observational HIV data allows for a robust evaluation of the antiretroviral treatment [ART] experience over time. For example, we have pooled data from multiple HIV clinical care sites to better understand outcomes of key populations (e.g., late ART initiators [10], older patients [11], and patients with 10+ years of follow-up [12]) as well as assess site-level progress in clinical retention, ART use, and viral suppression over time [13]. Other HIV cohorts have investigated a myriad of topics, including (but not limited to) efficacy and tolerability of ART regimens [14], comorbidities [15] and patient outcomes [16]. Findings from these investigations are communicated with researchers, local care providers, regional stakeholders, and global non-governmental organizations, and often influence public health policy decisions. Thus, it is critical to understand whether errors in the dataset lead to invalid inferences.

In this study, we assess the impact of SDV audits on results within a multi-cohort, international collaboration. External auditors traveled to nine sites and conducted SDV for all key HIV study variables on a randomly selected subset of patient records. After the audits, local sites received a report detailing audit findings and recommendations, which in certain cases included requests to re-enter error-prone variables for all patient records. In this manuscript, we perform analyses using data from the entire cohort, just before the audit and then two years after the audit, to investigate changes made to databases and the impact of the audit on key study findings.

## Methods

### Cohort description

The Caribbean, Central, and South America network for HIV epidemiology (CCASAnet) is a consortium of clinics from seven Latin American countries that collects and shares HIV care data. CCASAnet has been described elsewhere [17]; additional information is at https://www.ccasanet.org.

### Data auditing

In 2013–14, on-site audits of submitted data were conducted through a joint effort between data auditors from the CCASAnet Data Coordinating Center at Vanderbilt University (CDCC-VU) and investigators at nine participating sites. For each site, approximately 30 patient records were randomly selected to be audited. Source documents available at the sites included paper-based patient charts from the HIV clinic, general hospital charts, laboratory result forms (both paper and electronic), and electronic medical record systems.

An audit team from the CDCC-VU, consisting of at least one clinician and one informatician, traveled to each of the nine sites. The audit team had a paper audit form, prepared by the CDCC-VU data manager, displaying all submitted research data for each patient record selected. Over the course of 2–3 days, the data audit team compared values in the research database with the source documents. Additionally, the authors reviewed all available source documents to check whether values or entire visits that were present in the source documents were missing from the research database. Each entry was labeled with an audit code (A1-A5) adapted from standardized audit codes [18]: value matches source document (A1), discrepancy between database and source document (A2 if minor discrepancy, A3 if major), value in source document not previously entered in database (A4), and value could not be verified in source document (A5). New information identified from the source document (A2, A3, or A4) was noted on the paper audit form. All audit findings were later transcribed from the paper audit forms to a study database by the CDCC-VU. The original CCASAnet audit protocol and sample forms are available online [19].

In response to the audit, each site received scans of the audit forms and a report describing errors found and general recommendations. Site-specific advice included (but was not limited to) re-abstraction of ART regimens from older records, entry of missed visit or lab data that was available in the paper chart, more timely record updates for research data submissions, and more thorough collection of clinical events data.

### Available data

**Fig. 1:**
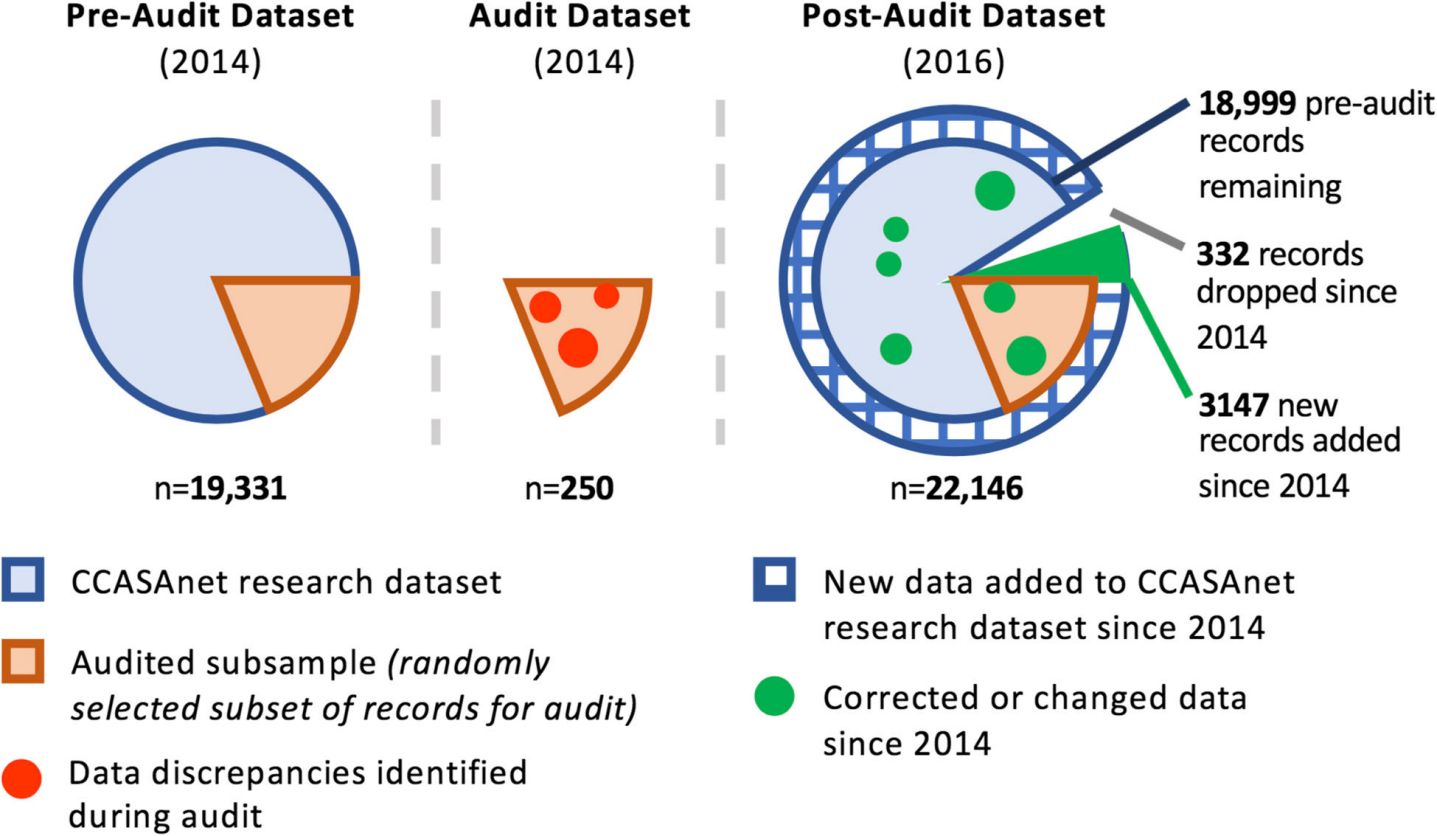
An overview of the CCASAnet data audit process

As part of routine CCASAnet collaboration, each site regularly submitted to the CDCC-VU a dataset containing records for all past and present enrolled patients. Prior to the audit, the most recent submission from each site was archived. These site-specific datasets were aggregated to generate a *pre-audit dataset*. Approximately two years after the audit (October 2016), the CDCC-VU again archived the most recent submission from each site and aggregated records for all patients to generate a *post-audit dataset*. This time frame encompassed 1–2 scheduled data submission cycles for each site, thereby allowing enough time for audit recommendations potentially to be incorporated in the new dataset. We note one key modification to the post-audit dataset: any data points after the site-specific pre-audit freeze date were removed from the post-audit dataset so that pre- and post- audit datasets covered the same time period. However, patient records not present in the pre-audit dataset but present in the post-audit dataset were included if the patient was enrolled prior to the pre-audit freeze date. Lastly, an *audited dataset* was generated for the subset of records that were audited. This dataset contained patient records according to the source document verification findings. The connection between all three datasets is shown visually in Fig. 1.

The three datasets each contained 21 variables (as defined and standardized in the CCASAnet data transfer protocol) that were routinely submitted by CCASAnet sites. Two variables (CDC and WHO stage at enrollment) were collected in the pre-audit and audited datasets, but were replaced with two different variables (a clinical AIDS indicator and the corresponding date of diagnosis) in the post-audit dataset; the remaining 19 were included in all three datasets. We refer to these variables as *primary variables*. We also generated 14 additional variables that were relevant for our statistical analyses. These *derived variables* were typically calculated using one or more of the primary variables (e.g., the CD4 cell count at time of ART initiation). A complete list of variables considered for this study is included in Additional file 1: Table S1.

### Statistical analysis

We defined a data discrepancy as an instance where recorded values were different or a value was missing in one of the two datasets. When comparing the audited dataset with the pre-audit dataset, we also counted instances where a value could not be verified as a discrepancy. We calculated discrepancy (error) rates for both the originally collected and derived variables used in analyses between (1) the pre-audit and audited datasets in the subset of records that were audited, and (2) the entire pre-audit and post-audit datasets.

To assess the impact of errors identified during a data audit on a typical statistical analysis, we replicated the same statistical analyses in all datasets. Patients were excluded if they were not adults (< 18 years) or never initiated ART. Two countries had multiple sites (Argentina and Honduras); for this analysis, we combined sites within a country into a single site. We estimated the overall and country-specific (when data were available) cumulative incidences for both the time from ART initiation to death and the time from ART initiation to first AIDS-defining event. A multivariable Cox regression model was fit to estimate cause-specific hazard ratios (HRs) for predictors of death and AIDS after ART initiation. All models were adjusted for the following covariates: age, sex, probable route of HIV infection, clinical history of AIDS, CD4 cell count, initial ART regimen, and calendar year. All Cox models were stratified by site to allow the underlying hazard to differ for each site [20] and used restricted cubic splines [21] with four knots for continuous variables to relax linearity assumptions. While we describe instances where the estimates were higher or lower and provided measures of uncertainty (i.e., 95% confidence intervals [95% CIs]), we did not test for statistical significance and avoid describing them as such.

Given that some patient records were included in only one dataset, we performed a sensitivity analyses that repeated the above-described analyses using only patient records that were available in both the pre-audit and post-audit dataset.

All analyses were performed using R Statistical Software (http://www.R-project.org); corresponding code is available at http://biostat.mc.vanderbilt.edu/ArchivedAnalyses. Institutional review board approval was obtained from each site and the CDCC-VU.

## Results

A total of 316 patient records from nine CCASAnet sites were selected to be audited using stratified random sampling by site. The CDCC-VU data auditors reviewed 250 (79%) of the selected records during the audit visits. The remaining 66 records were not audited, mainly due to insufficient time during the audit visits or unavailable source documents (including lost, accidentally destroyed, or permanently archived charts, and charts currently in use for patient care). The number of audited records varied by site, ranging from 12 to 31 (Additional file 1 Table S2).

### Audited records: pre-audit versus audit data

**Fig. 2:**
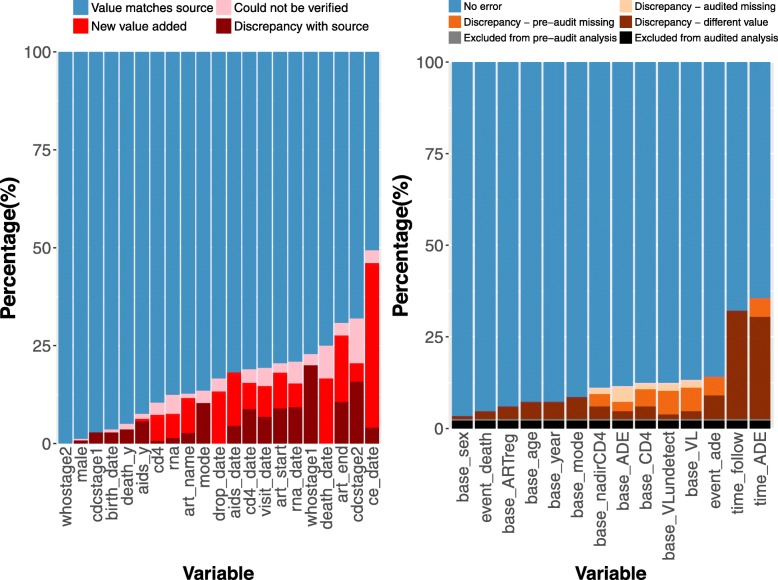
Relative frequency of discrepancies between pre-audit and audited values for originally collected variables and those derived for analysis

The pre-audit dataset for these 250 patients contained 19,289 values across 21 variables; 14,489 (75%) were audited due to time constraints and incomplete source documents. Overall, the discrepancy rate across all audited variables was 17.1% (*n* = 2480; Fig. 2a). Most discrepancies were due to missing values (*n* = 1066; 43%); the remaining were due to discrepant data entries (*n* = 843; 34%) and data that could not be verified (*n* = 571; 23%). Among variables typically collected at enrollment, error rates were low for sex (3/245; 1%) and birth date (9/246; 4%), and high for probable mode of infection (30/222; 14%). Only 5% (7/138) of patients had a discordant death status, yet approximately 25% (6/24) of all audited death dates had a discrepancy. Date variables had higher discrepancy rates, including 31% (133/431) for ART regimen end dates and 49% (120/243) for clinical event dates. Error rates for all audited variables are included in Additional file 1: Table S3 and Figure S1.

Of the 250 audited patients, 228 (91%) originally met inclusion criteria for analyses (adult patients who initiated ART) in the pre-audit dataset and 232 (93%) in the audited dataset; 227 (91%) met inclusion criteria in both datasets. Of the five patients excluded from the pre-audit dataset only, four had discrepancies in ART data and one was missing follow-up data. For the single patient excluded in the audited dataset only, a revised birthdate revealed the patient was under 18 at ART initiation. For records present in at least one dataset (*n* = 233), discrepancy rates for derived variables ranged from 3 to 36% (Fig. 2b). Variables with the highest error rates corresponded to derived time-to-event variables such as time from ART initiation to first AIDS-defining event (*n* = 83; 36%) and follow-up time (*n* = 75; 32%).

**Fig. 3:**
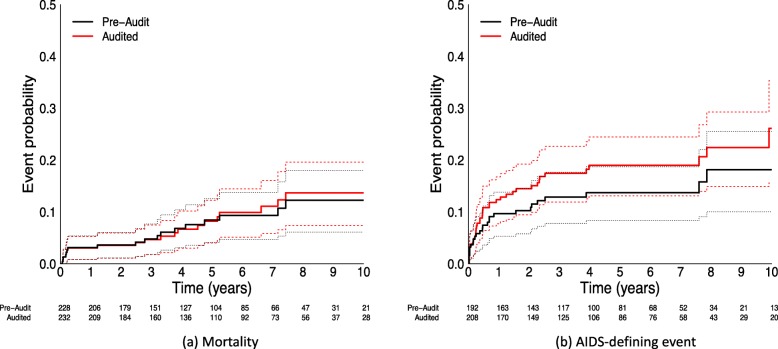
Unadjusted time to mortality (**a**) and AIDS-defining event (**b**) using pre-audit and audited data, among the subset of patient records that were audited. Solid lines denote the estimated incidence and dotted lines denote the corresponding 95% confidence intervals

Unadjusted estimates of mortality over time (Fig. 3a) were similar between audited patients in the pre-audit and audited datasets. Meanwhile, the overall estimated probability of AIDS over time was higher in the audit dataset (Fig. 3b). The estimated percentage of patients with an AIDS-defining event at three years was 12.9% (7.8, 17.6%) in the pre-audit dataset and 17.5% (11.9, 22.7%) in the audited dataset. Due to the small number of events among the subset of audited records, there was overlap in the confidence intervals for all hazard ratios (Additional file 1: Figure S2).

### Full dataset: pre-audit versus post-audit data

The full pre-audit database included 19,331 adult patients. The post-audit dataset, which incorporated sites’ data revisions in response to the audit findings, contained 22,146 eligible adult patients from the same time period (e.g., with enrollment dates prior to the site-specific freeze dates for the pre-audit dataset.) The post-audit revisions produced a dataset with 18,999 patients from the pre-audit dataset plus 3147 newly added patients. Some patients (*n* = 332) previously included in the pre-audit dataset were not present in the updated dataset; duplicate records or instances where the original paper forms could not be located were removed.

**Fig. 4:**
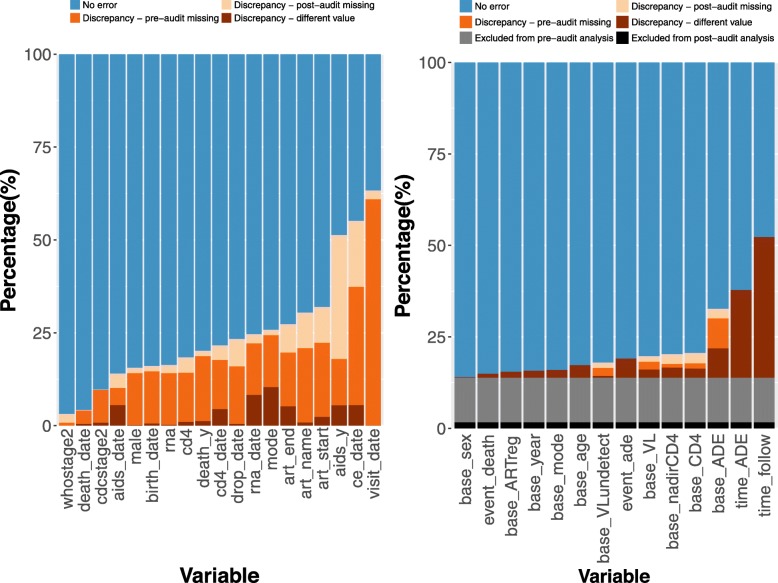
Relative frequency of discrepancies between pre-audit and post-audit values for originally collected variables and those derived for analysis among all records

For the 22,478 unique patients documented in one or both datasets, 1,884,334 unique fields were entered across 19 variables in either the pre-audit or post-audit dataset. Of these, 1,135,693 (60%) were identical in both datasets. The plurality (*n* = 624,414; 83%) of the discrepancies between the two datasets was due to missing values in the pre-audit dataset that were subsequently included in the post-audit dataset. Missing values in the post-audit dataset (*n* = 82,519) that existed in the pre-audit dataset explained 11% of discrepancies and conflicting values (*n* = 41,708) accounted for the remaining 6%. The variables with the highest proportion of discrepancies were prior history of AIDS at enrollment (11,544/22,478; 51%), the date of diagnosis of a clinical endpoint (6789 /12,309; 55%), and the date of clinic visit (420,688/664,269; 63%) (Fig. 4a). Discrepancy rates varied by site, ranging from 10 to 58%.

Overall, 17,703 patients were classified as adult ART initiators in at least one dataset. A total of 15,253 (86%) were in both the pre-audit and post-audit analysis cohorts and the remaining 2450 (14%) were discordant. Discrepancy rates for derived variables among the 17,703 patients that met inclusion criteria for at least one dataset ranged from 14% (*n* = 2480) for sex to 52% (*n* = 9265) for time from ART initiation to death or censoring (Fig. 4b).

**Fig. 5:**
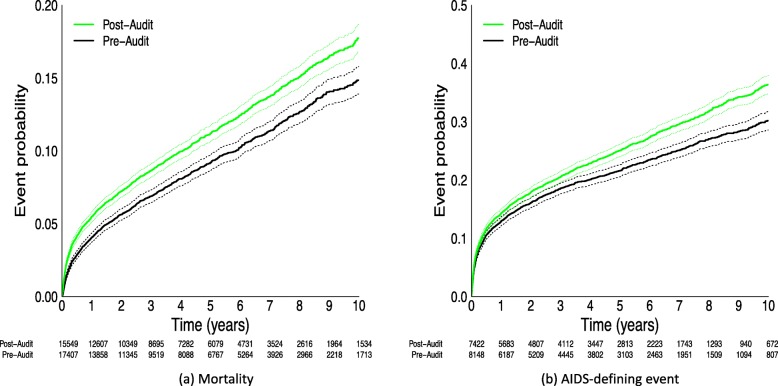
Unadjusted estimates of time to mortality (**a**) and AIDS-defining event (**b**) for patients in the pre-audit and post-audit datasets. Solid lines denote the estimated incidence and dotted lines denote the corresponding 95% confidence intervals

Estimated probabilities of mortality over time (Fig. 5a) were higher using the post-audit (*n* = 17,407) than the pre-audit (*n* = 15,549) dataset. The estimated percentage of patients who died by three years was 6.9% (95% CI: 6.4, 7.3%) in the pre-audit dataset and 8.7% (95% CI: 8.2, 9.1%) in the post-audit dataset. Using patient data from the five regions where clinical event data was available, estimated probabilities of an AIDS-defining event (Fig. 5b) were higher in the post-audit dataset (*n* = 8148) than the pre-audit dataset (*n* = 7422). The estimated percentage of patients with AIDS at three years was 18.6% (95% CI: 17.6, 19.5%) in the pre-audit dataset and 20.5% (95% CI: 19.6, 21.4%) in the post-audit dataset. Changes in mortality rates (Additional file 1: Figure S3) and AIDS-defining event rates (Additional file 1: Figure S4) varied by site. Two of the seven regions had similar mortality estimates; one had lower estimates and four had higher estimates using the post-audit dataset. AIDS estimates varied for all five regions with available data; estimates were higher for three sites and lower for two sites.

**Fig. 6:**
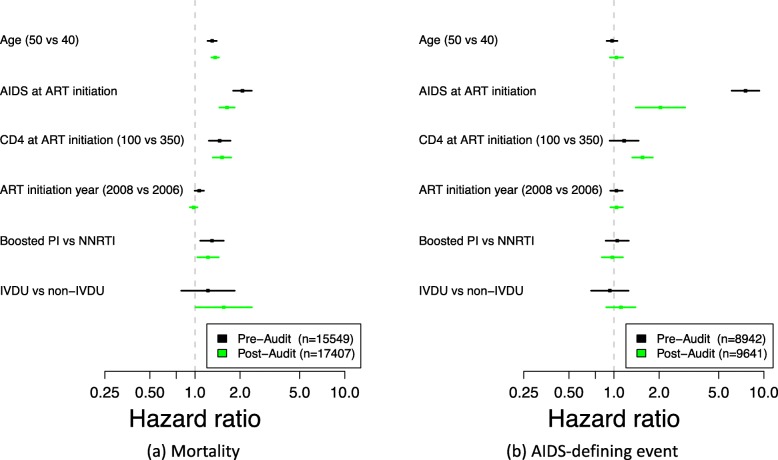
Adjusted hazard ratios of mortality (**a**) and AIDS-defining event (**b**) for patients in the pre-audit and post-audit datasets

In adjusted analyses, the hazard ratios corresponding to AIDS and mortality outcomes were shifted for some variables (Fig. 6 and Table 1). The hazard of death for patients with a prior history of clinical AIDS was lower in the post-audit dataset (HR: 1.64; 95% CI: 1.46, 1.84) than in the pre-audit dataset (HR: 2.07; 95% CI: 1.80, 2.39). The hazard of an AIDS-defining event for patients with a prior history of clinical AIDS was also lower in the post-audit dataset (HR:2.04; 95% CI: 1.40, 2.99) than in the pre-audit dataset (HR: 7.55; 95% CI: 6.10, 9.34). The hazard ratio of AIDS in the post-audit dataset relative to the pre-audit dataset was higher for patients with a lower CD4 cell count (1.55; 95% CI: 1.32, 1*.*82 vs. 1.17; 95% CI: 0.94, 1.46). Differences in the hazards of death (Additional file 1: Figure S5) and AIDS-defining events (Additional file 1: Figure S6) between pre-audit and post-audit datasets varied by site.

**Table 1:** Adjusted hazard ratios of mortality and AIDS-defining event for all patients enrolled at time of data audit using the pre-audit and post-audit datasets

	Mortality		AIDS-defining event	
	Pre-Audit Hazard Ratio	Post-Audit Hazard Ratio	Pre-Audit Hazard Ratio	Post-Audit Hazard Ratio
Gender				
Female	Ref	Ref	Ref	Ref
Male	1.08 (0.96, 1.21)	1.04 (0.94, 1.15)	0.88 (0.74, 1.04)	0.93 (0.84, 1.04)
Age				
20	1.11 (0.87, 1.42)	1.08 (0.87, 1.33)	1.18 (0.90, 1.54)	1.06 (0.86, 1.31)
30	0.92 (0.82, 1.03)	0.90 (0.82, 0.99)	1.05 (0.93, 1.19)	0.99 (0.89, 1.12)
40	Ref	Ref	Ref	Ref
50	1.30 (1.21, 1.40)	1.36 (1.29, 1.44)	0.97 (0.89, 1.05)	1.04 (0.94, 1.15)
60	1.77 (1.49, 2.11)	1.96 (1.71, 2.26)	0.94 (0.76, 1.17)	1.09 (0.85, 1.39)
Clinical AIDS at baseline				
No	Ref	Ref	Ref	Ref
Yes	2.07 (1.80, 2.39)	1.64 (1.46, 1.84)	7.55 (6.10, 9.34)	2.04 (1.40, 2.99)
Nadir CD4				
50	1.90 (1.59, 2.27)	1.96 (1.68, 2.28)	1.31 (1.02, 1.70)	1.87 (1.56, 2.25)
100	1.46 (1.24, 1.73)	1.52 (1.32, 1.75)	1.17 (0.94, 1.46)	1.55 (1.32, 1.82)
200	1.05 (0.92, 1.19)	1.11 (0.99, 1.23)	0.98 (0.86, 1.13)	1.14 (1.04, 1.24)
350	Ref	Ref	Ref	Ref
Initiation Year				
2000	0.83 (0.70, 0.99)	0.91 (0.79, 1.06)	1.00 (0.83, 1.22)	1.15 (0.92, 1.43)
2002	0.85 (0.74, 0.97)	1.01 (0.91, 1.13)	0.94 (0.79, 1.13)	1.08 (0.87, 1.34)
2004	0.90 (0.82, 0.99)	1.05 (0.97, 1.13)	0.95 (0.87, 1.05)	1.02 (0.90, 1.17)
2006	Ref	Ref	Ref	Ref
2008	1.07 (1.00, 1.15)	0.98 (0.93, 1.04)	1.04 (0.94, 1.14)	1.04 (0.95, 1.14)
2010	1.03 (0.84, 1.25)	1.08 (0.93, 1.26)	1.06 (0.85, 1.33)	1.15 (0.96, 1.39)
2012	0.94 (0.62, 1.42)	1.27 (0.92, 1.74)	1.09 (0.74, 1.59)	1.30 (0.96, 1.77)
ARV CLASS				
NNRTI	Ref	Ref	Ref	Ref
Boosted PI	1.30 (1.08, 1.55)	1.22 (1.04, 1.44)	1.05 (0.88, 1.25)	0.97 (0.83, 1.15)
Other	1.11 (0.91, 1.37)	1.21 (1.02, 1.44)	0.99 (0.80, 1.24)	1.14 (0.88, 1.48)
IVDU				
No	Ref	Ref	Ref	Ref
Yes	1.22 (0.81, 1.84)	1.56 (1.01, 2.40)	0.94 (0.70, 1.25)	1.11 (0.89, 1.39)

As a sensitivity analysis, we compared the pre-audit and post-audit datasets after removing patient records that were not present in both the pre-audit and the post-audit dataset as well as data points in the post-audit dataset that occurred after the last patient’s pre-audit date. For the 18,999 patients in both databases, 1,727,710 unique values were recorded; 1,135,693 (66%) were identical. The variables with the highest proportion of entries with discrepancies were similar to the full dataset comparison: the date of diagnosis of a clinical endpoint (51%), prior history of AIDS at enrollment (52%) and the date of clinic visit (60%). Among this cohort of 18,999 patients, 15,441 patients met inclusion criteria for at least one analysis dataset. Discrepancy rates for derived variables ranged from 2% for sex to 23% for clinical AIDS status at baseline. Most variables had a lower relative frequency of discrepancies in the post-audit dataset compared to the error rates from the audited subset of records alone. The key exception was occurrence of an AIDS-defining event at baseline (23% vs. 12%). While the estimated percentage of patients with an AIDS-defining event at three years was still higher in the post-audit dataset (20.9% vs. 18.6%), mortality estimates over time were similar using the pre-audit and post-audit datasets.

## Discussion

This study demonstrated that results and corresponding inferences can be affected by improvements in data quality following data audits. Source data verification in our multi-region observational HIV cohort revealed substantial errors in the recording of certain variables, in particular clinical events. Recommendations by audit teams led to re-entry of some variables at some sites. Subsequent analyses of revised, post-audit datasets yielded results consistent with audit findings. In particular, rates of AIDS-defining events after ART initiation were substantially higher in audit data than in pre-audit data, and ensuing analyses using post-audit data similarly estimated rates of an AIDS-defining event to be much higher than those observed pre-audit. These results suggest that the quality of clinical events data improved as a result of the audit.

There are many possible reasons for data errors, ranging from isolated errors such as typographical mistakes and misread values due to illegible handwriting to systematic issues such as misinterpreted variable definitions, miscoded value sets, or mistakes in assembling databases. For example, high error rates in dates of CD4 and viral load measurements discovered during an audit at one site uncovered a systematic error in how data entry personnel had been trained to enter this data into the study database. As a result of the audit, investigators at that site were made aware of the issue and were able to fix existing errors and prevent future invalid entries. For a multi-site consortium, early identification and rapid resolution of systematic issues can have a profound impact on data quality.

A previous CCASAnet audit was conducted in 2008–2009. However, this was the first time that the clinical endpoints data were audited. Error rates for these variables tended to be higher than those for variables that had been previously audited. Clinical endpoint entries may be particularly prone to error and improper extraction by data capture personnel who lack the necessary clinical background to identify diagnoses from paper charts. However, the high error rates in clinical endpoints variables may also be indirect evidence that the audit process worked: variables that have been previously audited could be less likely to be error-prone in the next wave of audits because major errors have been identified and causes recognized and fixed.

Our findings suggest that variable modification (e.g., replacing missing values or amending a previous entry) rates for most variables were higher in the audit database than in the post-audit database. This is not surprising: variables with low audit-determined error rates would remain largely unchanged in post-audit databases. However, it does serve as a reminder that when conducted on a random subset of records, data audits mostly improve data quality among all patients for specific variables with systematic issues and to a lesser extent the remaining variables among the audited patients. In ongoing work, we are considering statistical methods that can use audit data to predict errors for unaudited patients and thus improve analyses using error-prone data [22].

Discrepancy rates tended to be lower for derived variables than for primary variables. Given that derived variables are typically composed of two or more primary variables, we had anticipated that they would be more error-prone. A closer review reveals that a large number of discrepancies in the primary variables were due to missingness. For variables that were routinely collected at each visit, a missing entry was often inconsequential when generating analysis variables as derived variables were often calculated using windows that include multiple visits. This reaffirms that the impact of questionable data on study findings is difficult to assess by only using error rates [8, 9].

While source data verification is frequently used to monitor data quality in a clinical setting, there is little consensus on best practices for implementing and assessing such audits. A recent review of 15 published studies regarding SDV found a wide range of approaches with no standard method of evaluation [23]. Our findings suggest that, as investigators move to standardize SDV practices, data quality evaluations should focus on key variables likely to be included in statistical analyses.

Our study has limitations. Most notably, our study design did not allow us to differentiate between improvements in data quality due to the audit process and natural improvements in data over time. We recognize that some changes (e.g., entry of backlog visits) may have occurred independently from the audit process. In addition, there is no gold standard, and some audit data may not reflect the patient’s reality.

## Conclusions

The SDV process can improve data quality, which can in turn have an impact on epidemiological inferences, especially for variables like the CCASAnet clinical endpoints data that had not been audited previously. We encourage the implementation of data audits for observational studies that rely on the extraction of study data from source documents.

## Supplementary information

**Additional file 1: Table S1.** Complete list of study variables with descriptions. **Table S2.** Overview of audit frequency by site. **Figure S1.** Summary of reported audit findings for all audited variables. **Table S3.** Auditing results for each variable entry. **Figure S2.** Adjusted hazard ratios of mortality (a) and AIDS-defining event (b) for patients in the pre-audit and audited datasets. **Figure S3.** Estimated cumulative incidence of death by site for patients in the pre-audit and post-audit datasets. Solid lines denote the estimated incidence and dotted lines denote the corresponding 95% confidence intervals. **Figure S4.** Estimated cumulative incidence of an AIDS-defining event by site for patients in the pre-audit and post-audit datasets. Solid lines denote the estimated incidence and dotted lines denote the corresponding 95% confidence intervals. **Figure S5.** Association between patient characteristics at baseline and hazard of death by site using pre-audit and post-audit data. The dots denote the estimated incidence and the lines denote the corresponding 95% confidence intervals. **Figure S6**. Association between patient characteristics at baseline and hazard of an AIDS-defining event by site using pre-audit and post-audit data. The dots denote the estimated incidence and the lines denote the corresponding 95% confidence intervals.

## Data Availability

Complete data for this study cannot be publicly shared because of legal and ethical restrictions. The Principles of Collaboration under which the CCASAnet multi-national collaboration was founded and the regulatory requirements of the different countries’ IRBs require the submission and approval of a project concept sheet by the CCASAnet Executive Committee and the principal investigators at participating sites. All datasets provided by CCASAnet are de-identified according to HIPAA Safe Harbor guidelines. CCASAnet promotes the signing of a Data Use Agreement before HIV clinical data can be released. Instructions for how to obtain CCASAnet data are outlined on the CCASAnet website: https://www.ccasanet.org/collaborate/.

## Abbreviations

CCASAnet: The Caribbean, Central, and South America network for HIV epidemiology
CDCC-VU: CCASAnet Data Coordinating Center at Vanderbilt University
SDV: Source document verification

## References

[1] Weiskopf NG, Weng C (2013). Methods and dimensions of electronic health record data quality assessment: enabling reuse for clinical research. J Am Med Inform Assoc.

[2] Kiragga AN, Castelnuovo B, Schaefer P, Muwonge T, Easterbrook PJ (2011). Quality of data collection in a large HIV observational clinic database in sub-Saharan Africa: implications for clinical research and audit of care. J Int AIDS Soc.

[3] Nicol E, Dudley L, Bradshaw D (2016). Assessing the quality of routine data for the prevention of mother-to-child transmission of HIV: an analytical observational study in two health districts with high HIV prevalence in South Africa. Int J Med Inform.

[4] Muthee V, Bochner AF, Osterman A, Liku N, Akhwale W, Kwach J, Prachi M, Wamicwe J, Odhiambo J, Onyango F, Puttkammer N (2018). The impact of routine data quality assessments on electronic medical record data quality in Kenya. PLoS One.

[5] Puttkammer N, Baseman JG, Devine EB, Valles JS, Hyppolite N, Garilus F, Honoré JG, Matheson AI, Zeliadt S, Yuhas K, Sherr K (2016). An assessment of data quality in a multi-site electronic medical record system in Haiti. Int J Med Inform.

[6] Duda SN, Shepherd BE, Gadd CS, Masys DR, McGowan CC (2012). Measuring the quality of observational study data in an international HIV research network. PLoS One.

[7] Houston L, Probst Y, Humphries A (2015). Measuring data quality through a source data verification audit in a clinical research setting. Stud Health Technol Inform.

[8] Mitchel JT, Kim YJ, Choi J, Park G, Cappi S, Horn D, Kist M, D'Agostino RB (2011). Evaluation of data entry errors and data changes to an electronic data capture clinical trial database. Drug information journal.

[9] Smith CT, Stocken DD, Dunn J, Cox T, Ghaneh P, Cunningham D, Neoptolemos JP (2012). The value of source data verification in a cancer clinical trial. PLoS One.

[10] Crabtree-Ramírez B, Caro-Vega Y, Shepherd BE, Wehbe F, Cesar C, Cortés C, Padgett D, Koenig S, Gotuzzo E, Cahn P, McGowan C (2011). Cross-sectional analysis of late HAART initiation in Latin America and the Caribbean: late testers and late presenters. PLoS One.

[11] Carriquiry G, Giganti MJ, Castilho JL, Jayathilake K, Cahn P, Grinsztejn B, Cortes C, Pape JW, Padgett D, Sierra-Madero J, McGowan CC (2018). Virologic failure and mortality in older ART initiators in a multisite Latin American and Caribbean cohort. J Int AIDS Soc.

[12] Wolff MJ, Giganti MJ, Cortes CP, Cahn P, Grinsztejn B, Pape JW, Padgett D, Sierra-Madero J, Gotuzzo E, Duda SN, McGowan CC (2017). A decade of HAART in Latin America: long term outcomes among the first wave of HIV patients to receive combination therapy. PLoS One.

[13] Rebeiro PF, Cesar C, Shepherd BE, De Boni RB, Cortés CP, Rodriguez F, Belaunzarán-Zamudio P, Pape JW, Padgett D, Hoces D, McGowan CC (2016). Assessing the HIV care continuum in Latin America: progress in clinical retention, cART use and viral suppression. J Int AIDS Soc.

[14] Wandeler G, Gerber F, Rohr J, Chi BH, Orrell C, Chimbetete C, Prozesky H, Boulle A, Hoffmann CJ, Gsponer T, Fox MP (2014). Tenofovir or zidovudine in second-line antiretroviral therapy after stavudine failure in southern Africa. Antivir Ther.

[15] Ahn MY, Jiamsakul A, Khusuwan S, Khol V, Pham TT, Chaiwarith R, Avihingsanon A, Kumarasamy N, Wong WW, Kiertiburanakul S, Pujari S (2019). The influence of age-associated comorbidities on responses to combination antiretroviral therapy in older people living with HIV. J Int AIDS Soc.

[16] Jiamsakul A, Kiertiburanakul S, Ng OT, Chaiwarith R, Wong W, Ditangco R, Nguyen KV, Avihingsanon A, Pujari S, Do CD, Lee MP. Long-term loss to follow-up in the TREAT Asia HIV observational database (TAHOD). HIV medicine. 2019;20(7):439–49. 10.1111/hiv.12734 PMC6639129 30980495

[17] McGowan CC, Cahn P, Gotuzzo E, Padgett D, Pape JW, Wolff M, Schechter M, Masys DR (2007). Cohort profile: Caribbean, central and South America network for HIV research (CCASAnet) collaboration within the international epidemiologic databases to evaluate AIDS (IeDEA) programme. Int J Epidemiol.

[18] Vantongelen K, Rotmensz N, Van Der Schueren E (1989). Quality control of validity of data collected in clinical trials. Eur J Cancer.

[19] Duda S, McGowan C, Wehbe F, Masys D (2008). White paper: The CCASAnet Data Audit Process. Distributed to the IeDEA network July 2008.

[20] Giganti MJ, Luz PM, Caro-Vega Y, Cesar C, Padgett D, Koenig S, Echevarria J, McGowan CC, Shepherd BE (2015). A comparison of seven Cox regression-based models to account for heterogeneity across multiple HIV treatment cohorts in Latin America and the Caribbean. AIDS Res Hum Retrovir.

[21] Shepherd BE, Rebeiro PF (2017). Assessing and interpreting the association between continuous covariates and outcomes in observational studies of HIV using splines. J Acquir Immune Defic Syndr.

[22] Shepherd BE, Yu C (2011). Accounting for data errors discovered from an audit in multiple linear regression. Biometrics..

[23] Houston L, Probst Y, Martin A (2018). Assessing data quality and the variability of source data verification auditing methods in clinical research settings. J Biomed Inform.

