# Supplementary material for: The impact of data quality and source data verification on epidemiologic inference: a practical application using HIV observational data
Source: BMC Public Health. 2019 Dec 30;19:1748. doi: 10.1186/s12889-019-8105-2 (PMC6937856; doi:10.1186/s12889-019-8105-2)
Supplement: Supplementary file 1 — Additional file 1: Table S1. Complete list of study variables with descriptions. Table S2. Overview of audit frequency by site. Figure S1. Summary of reported audit findings for all audited variables. Table S3. Auditing results for each variable entry. Figure S2. Adjusted hazard ratios of mortality (a) and AIDS-defining event (b) for patients in the pre-audit and audited datasets. Figure S3. Estimated cumulative incidence of death by site for patients in the pre-audit and post-audit datasets. Solid lines denote the estimated incidence and dotted lines denote the corresponding 95% confidence intervals. Figure S4. Estimated cumulative incidence of an AIDS-defining event by site for patients in the pre-audit and post-audit datasets. Solid lines denote the estimated incidence and dotted lines denote the corresponding 95% confidence intervals. Figure S5. Association between patient characteristics at baseline and hazard of death by site using pre-audit and post-audit data. The dots denote the estimated incidence and the lines denote the corresponding 95% confidence intervals. Figure S6. Association between patient characteristics at baseline and hazard of an AIDS-defining event by site using pre-audit and post-audit data. The dots denote the estimated incidence and the lines denote the corresponding 95% confidence intervals. [file 12889_2019_8105_MOESM1_ESM.pdf]

Supplemental Tables and Figures for The impact of data quality and source data verification on epidemiologic inference: a practical application using HIV observational data

Supplemental Table 1: Complete list of study variables with descriptions.

Variable	Description	Source	Dataset			Variable Audited
			Pre-Audit	Audited	Post-Audit	in 2008-09
birth_date	Birth date	tblBasic	Yes	Yes	Yes	Yes
male	Gender at birth (0-Female, 1-Male, 9-Unknown)	tblBasic	Yes	Yes	Yes	Yes
mode	Probable Mode of Infection	tblBasic	Yes	Yes	Yes	Yes
aids_y	AIDS dx before 1st visit	tblBasic	Yes	Yes	Yes	No
aids_date	AIDS dx date (if *aids_y*=1)	tblBasic	Yes	Yes	Yes	No
cdcstage [a]	CDC Stage at enrollment	tblBasic	Yes	Yes	No	Yes
whostage [a]	WHO Stage at enrollment	tblBasic	Yes	Yes	No	Yes
aids_cl_y [a]	Clinical AIDS dx before 1st visit	tblBasic	No	No	Yes	No
aids_cl_date [a]	Clinical AIDS dx date (if *aids_y*=1)	tblBasic	No	No	Yes	No
death_y	Did patient pass away?	tblFollow	Yes	Yes	Yes	Yes
death_date	Death date (if *death_y*=1)	tblFollow	Yes	Yes	Yes	Yes
drop_date	Date patient dropped from cohort	tblFollow	Yes	Yes	Yes	No
visit_date	Clinical encounter date	tblVisit	Yes	Yes	Yes	Yes
cdcstage	CDC Stage	tblVisit	Yes	Yes	Yes	No
whostage	WHO Stage	tblVisit	Yes	Yes	Yes	No
cd4_date	Date of CD4 lab test	tblLab_CD4	Yes	Yes	Yes	Yes
cd4	CD4 count value	tblLab_CD4	Yes	Yes	Yes	Yes
rna_date	Date of RNA lab test	tblLab_RNA	Yes	Yes	Yes	Yes
rna	RNA value	tblLab_RNA	Yes	Yes	Yes	Yes
art_start	Date of ART drug start	tblART	Yes	Yes	Yes	Yes
art_end	Date of ART drug end	tblART	Yes	Yes	Yes	Yes
art_name	Code representing ART drug(s)	tblART	Yes	Yes	Yes	Yes
ce_date	Date of clinical outcome	tblCE	Yes	Yes	Yes	No
base_sex	Gender at birth (0-Female, 1-Male, 9-Unknown)	Derived	Yes	Yes	Yes	
base_mode	Probable Mode of Infection	Derived	Yes	Yes	Yes	
base_year	Year of ART initiation	Derived	Yes	Yes	Yes	
base_age	Age at ART initiation	Derived	Yes	Yes	Yes	
base_ADE	Clinical AIDS at ART initiation	Derived	Yes	Yes	Yes	
base_ARTregimen	1st ART regimen	Derived	Yes	Yes	Yes	
base_CD4	CD4 cell count at ART initiation	Derived	Yes	Yes	Yes	
base_nadirCD4	Lowest CD4 cell count prior to ART initiation	Derived	Yes	Yes	Yes	
base_VL	Viral load at ART initiation	Derived	Yes	Yes	Yes	
base_VLundetectable	Undetectable VL at ART initiation	Derived	Yes	Yes	Yes	
event_death	Did patient pass away?	Derived	Yes	Yes	Yes	
event_ADE	Did patient have post-ART clinical outcome?	Derived	Yes	Yes	Yes	
time_follow	Time from ART initiation to death/censoring	Derived	Yes	Yes	Yes	
time_ADE	Time from ART initiation to ADE/censoring	Derived	Yes	Yes	Yes	

The data protocol further defining variables is available at https://www.ccasanet.org/resources/.

[a] In 2014, the data transfer protocol pertaining to clinical AIDS status at enrollment was updated. Certain variables were deprecated in tblBasic and replaced with new variables. These variables are not included in comparisons of pre-audit and post-audit datasets.

Supplemental Table 2: Overview of audit frequency by site.

Site	Audited N(%)	Not Audited N(%)	Total
Site A	31 (89%)	4 (11%)	35
Site B	12 (57%)	9 (43%)	21
Site C	31 (52%)	29 (48%)	60
Site D	34 (97%)	1 (3%)	35
Site E	28 (80%)	7 (20%)	35
Site F	30 (100%)	0 (0%)	30
Site G	30 (100%)	0 (0%)	30
Site H	24 (69%)	11(31%)	35
Site I	30 (86%)	5 (14%)	35
Overall	250	66	316

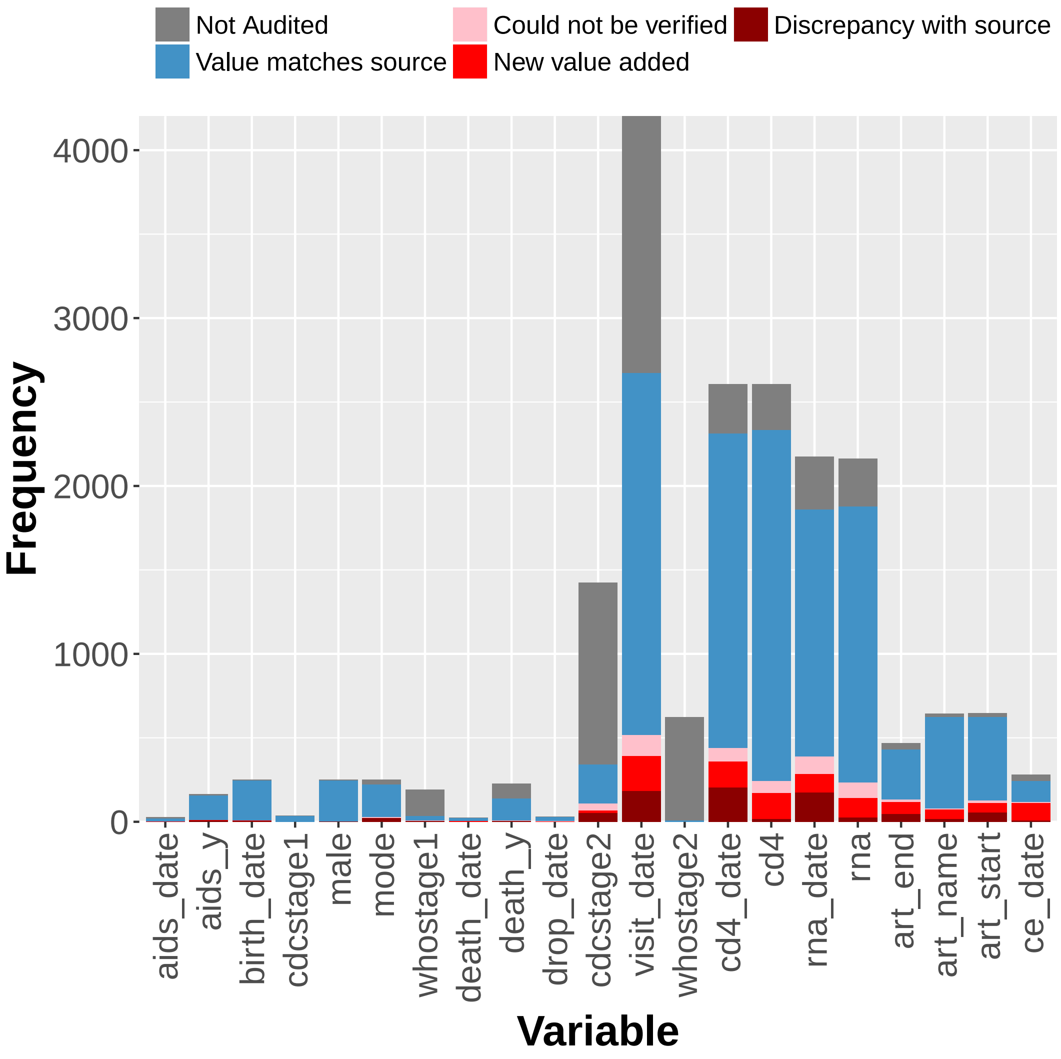

Supplemental Figure 1: Summary of reported audit findings for all audited variables.

Supplemental Table 3: Auditing results for each variable entry.

Form	Variable	Total Entries	Not audited	Total Audited	Value matches source A1	Discrepancy with source A2 + A3	New value added A4	Could not be verified A5	Error Rate (A2+A3+A4+A5)/ (A1+A2+A3+A4+A5)
basic									
	male	250	5	245	242	2	0	1	1.2%
	cdcstage1	35	0	35	34	1	0	0	2.9%
	birth.date	250	4	246	237	7	0	2	3.7%
	aids.y	164	6	158	146	9	1	2	7.6%
	mode	250	28	222	192	23	0	7	13.5%
	aids.date	28	6	22	18	1	3	0	18.2%
	whostage1	192	157	35	27	7	0	1	22.9%
follow									
	death.y	226	88	138	131	5	0	2	5.1%
	drop.date	30	0	30	25	0	4	1	16.7%
	death.date	26	2	24	18	0	4	2	25.0%
visit									
	whostage2	623	616	7	7	0	0	0	0.0%
	visit.date	4203	1529	2674	2157	183	211	123	19.3%
	cdcstage2	1425	1084	341	232	54	16	39	32.0%
cd4									
	cd4	2607	273	2334	2090	17	155	72	10.5%
	cd4.date	2607	292	2315	1875	205	155	80	19.0%
rna									
	rna.v	2163	285	1878	1644	27	116	91	12.5%
	rna.date	2175	314	1861	1471	173	113	104	21.0%
art									
	art.name	644	18	626	546	17	56	7	12.8%
	art.start	645	21	624	496	56	57	15	20.5%
	art.end	467	36	431	298	46	73	14	30.9%
ce									
	ce.date	279	36	243	123	10	102	8	49.4%
	overall	19289	4800	14489	12009	843	1066	571	17.1%

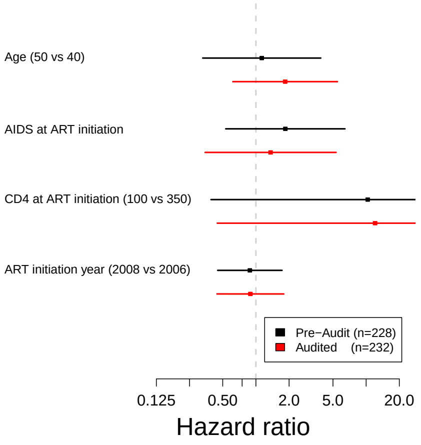

(a) Mortality

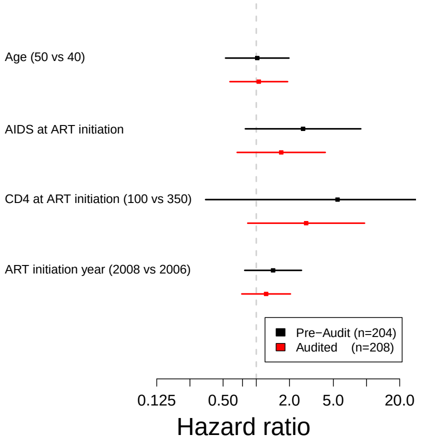

(b) AIDS-defining event

Supplemental Figure 2: Adjusted hazard ratios of mortality (a) and AIDS-defining event (b) for patients in the pre-audit and audited datasets.

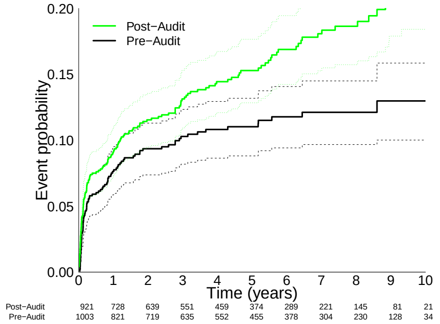

(a) Site 1

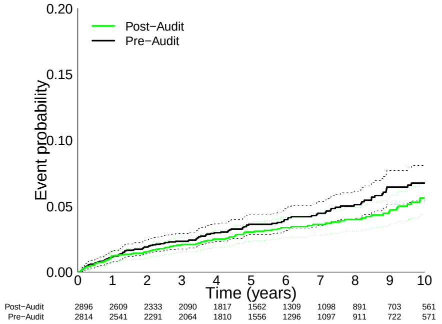

(b) Site 2

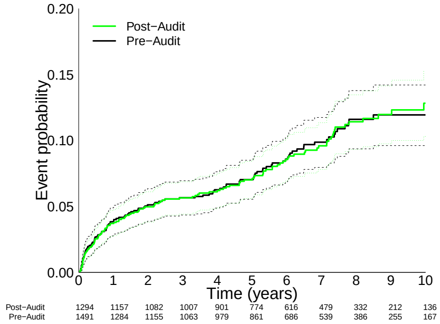

(c) Site 3

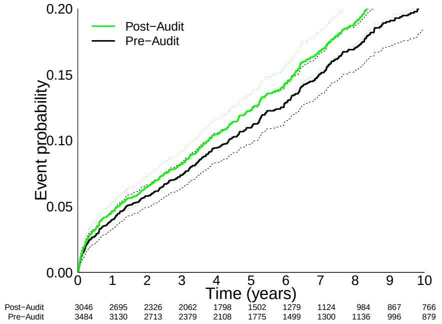

(d) Site 4

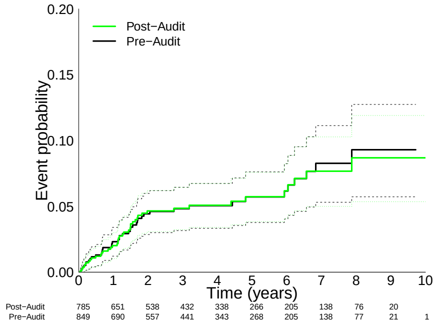

(e) Site 5

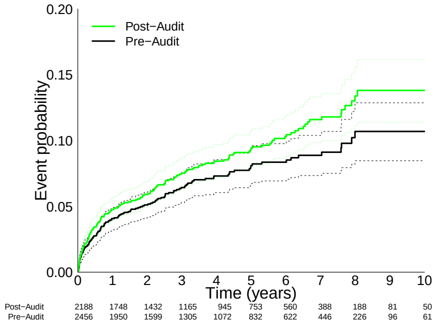

(f) Site 6

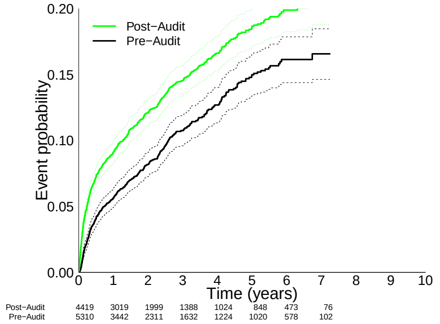

(g) Site 7

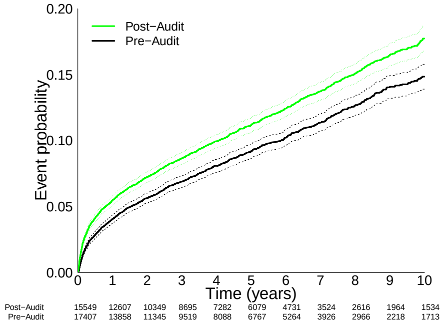

(h) Overall

Supplemental Figure 3: Estimated cumulative incidence of death by site for patients in the pre-audit and post-audit datasets. Solid lines denote the estimated incidence and dotted lines denote the corresponding 95% confidence intervals.

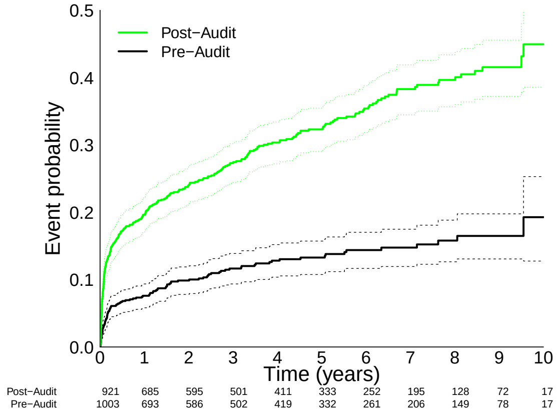

(a) Site 1

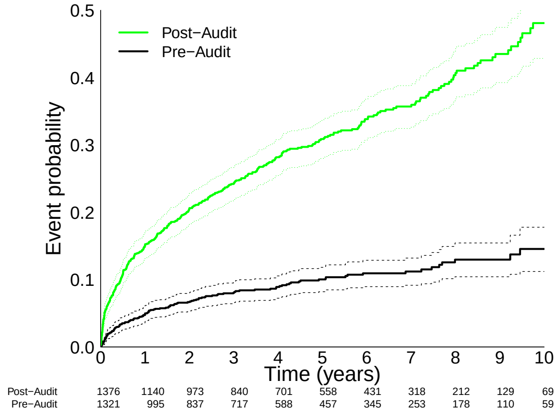

(b) Site 2

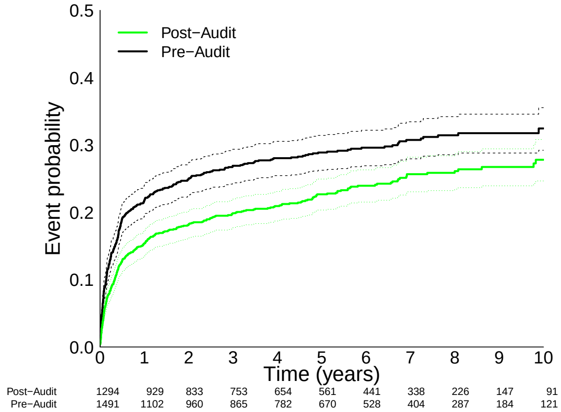

(c) Site 3

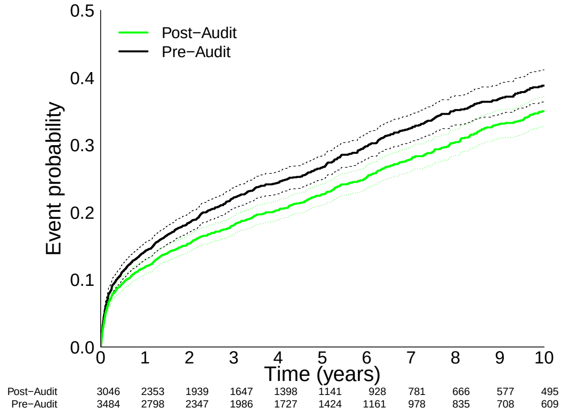

(d) Site 4

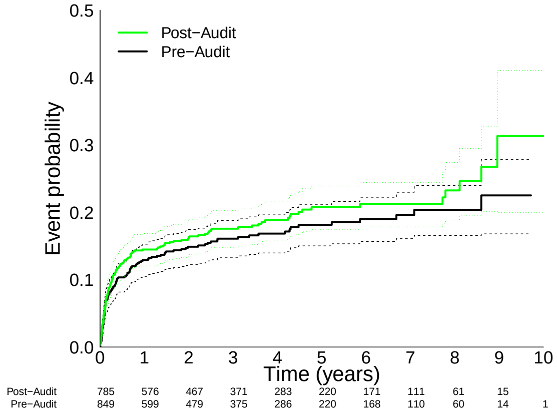

(e) Site 5

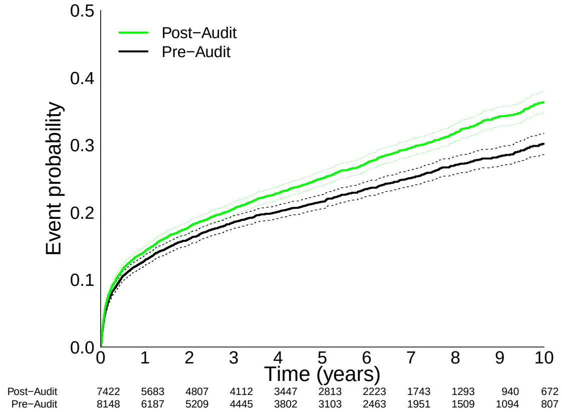

(f) Overall

Supplemental Figure 4: Estimated cumulative incidence of an AIDS-defining event by site for patients in the pre-audit and post-audit datasets. Solid lines denote the estimated incidence and dotted lines denote the corresponding 95% confidence intervals.

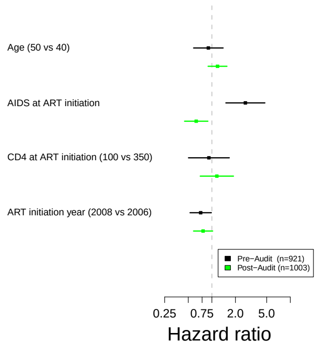

(a) Site 1

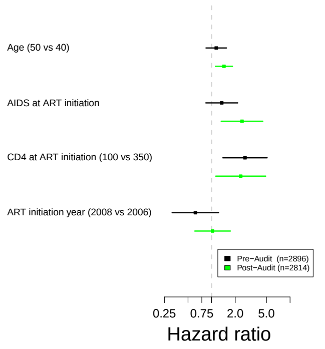

(b) Site 2

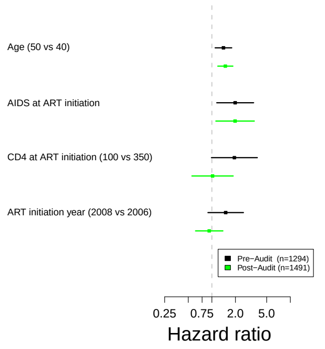

(c) Site 3

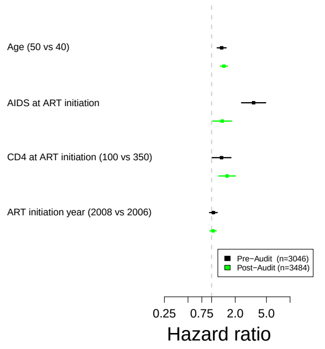

(d) Site 4

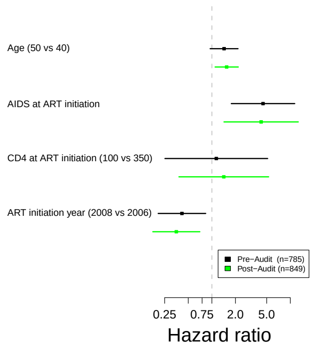

(e) Site 5

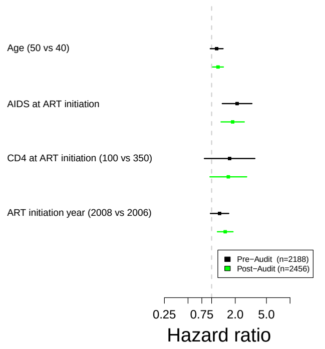

(f) Site 6

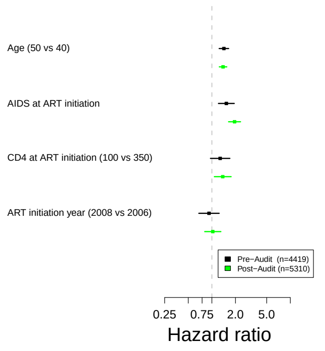

(g) Site 7

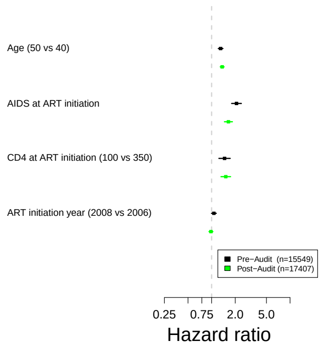

(h) Overall

Supplemental Figure 5: Association between patient characteristics at baseline and hazard of death by site using pre-audit and post-audit data. The dots denote the estimated incidence and the lines denote the corresponding 95% confidence intervals.

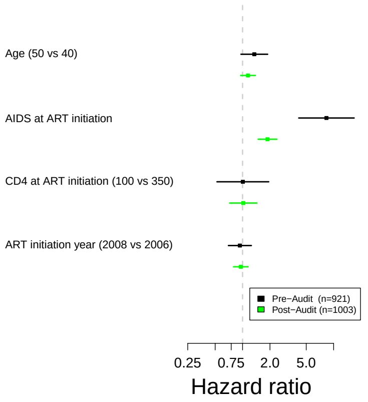

(a) Site 1

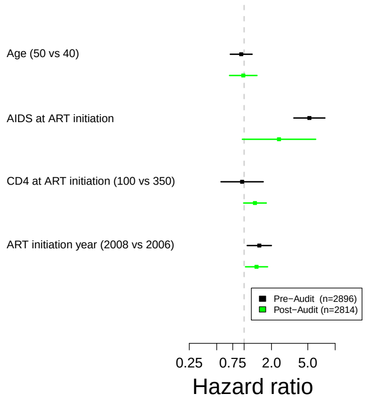

(b) Site 2

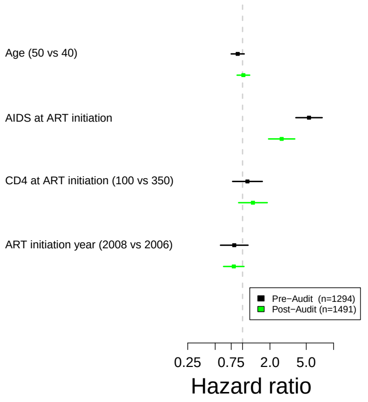

(c) Site 3

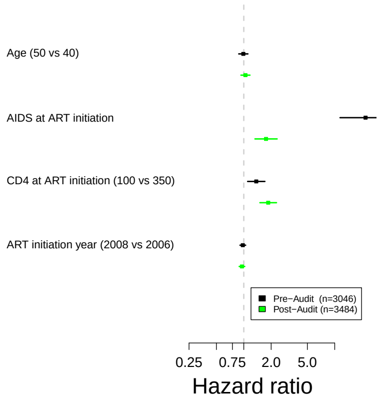

(d) Site 4

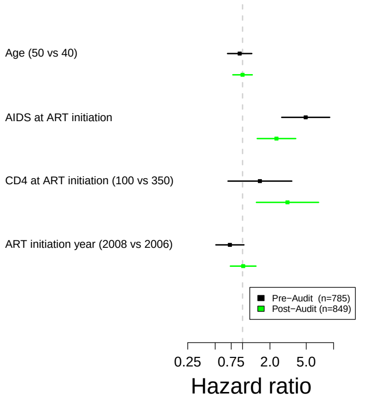

(e) Site 5

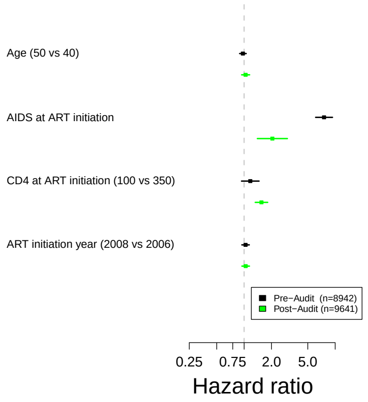

(f) Overall

Supplemental Figure 6: Association between patient characteristics at baseline and hazard of an AIDS-defining event by site using pre-audit and post-audit data. The dots denote the estimated incidence and the lines denote the corresponding 95% confidence intervals.
